# Supplementary material for: Harvesting easter eggs: An exploratory study of enjoying transnarrative media
Source: PLoS One. 2026 Feb 2;21(2):e0341588. doi: 10.1371/journal.pone.0341588 (PMC12863685; doi:10.1371/journal.pone.0341588)
Supplement: Attachment — Submitted filename: refree1.docx [file pone.0341588.s001.docx]

The paper titled "Study of Enjoying Transnarrative Media." This paper investigates the concept of transnarrative media and how audiences engage with and enjoy these narratives. It aims to explore the characteristics of transnarrative media and the factors influencing audience enjoyment.

**Summary of Key Points**

The authors present a thoughtful examination of transnarrative media, emphasizing the role of audience engagement. However, several areas need improvement to enhance the clarity and depth of the research.

**Strengths**

1. **Innovative Topic:** The exploration of transnarrative media is both timely and relevant, particularly in the context of evolving media consumption patterns.
2. **Theoretical Framework:** The application of Self-Determination Theory (SDT) is a solid foundation for understanding audience motivation and enjoyment.

**Areas for Improvement**

1. **Comprehensive Literature Review:** The paper requires a more extensive literature review, particularly incorporating recent studies that address transnarrative media and audience engagement. This will provide a stronger theoretical context for the research.
2. **Integration of Additional Theories:** While the authors utilize Self-Determination Theory, it would be beneficial to mention and discuss competitive theories such as Social Comparison Theory, Goal Orientation Theory, Achievement Goal Theory, and Self-Efficacy Theory. Integrating these theories can provide a more nuanced understanding of how audiences interpret their experiences with transnarrative media.
3. **Audience Behaviors:** The authors briefly touch on audience behaviors; however, there is a need for a more comprehensive explanation of the various interactions and drivers that impact audience behavior. A detailed exploration of these drivers will enhance the understanding of audience engagement and experience.
4. **Mental Models and Limitations:** The authors state, “In the current study, we propose that the ability to construct mental models of transnarrative media results in enjoyment and an intrinsically rewarding experience.” It is essential to address the limitations associated with mental models, including:
   - **Simplification of Reality**
   - **Bias and Subjectivity**
   - **Rigidity (Resistance to Change)**
   - **Cognitive Overload**

Acknowledging these limitations will provide a more balanced perspective and enhance the credibility of the research.

5. **Participant Characteristics:** The paper should better address the characteristics of the participants, particularly their range of ages and experiences. This information is crucial for understanding how these factors may influence audience engagement and enjoyment.
