# Supplementary material for: Harvesting easter eggs: An exploratory study of enjoying transnarrative media
Source: PLoS One. 2026 Feb 2;21(2):e0341588. doi: 10.1371/journal.pone.0341588 (PMC12863685; doi:10.1371/journal.pone.0341588)
Supplement: Attachment — Submitted filename: Response to Reviewers.docx [file pone.0341588.s002.docx]

*Dear Dr. Ahmadinia and Reviewers, we appreciate the opportunity to revise our study, “Harvesting Easter Eggs: An Exploratory Study of Enjoying Transnarrative Media” and have incorporated the reviewer feedback into our revised manuscript. Please see our comments below. Revisions are denoted with blue font in the manuscript. We highlight here the major changes made on this manuscript:*

- *We added supporting literature relevant to our arguments about SDT, mental models, and the tripartite model.*
- *We reran our analyses to include easter egg recall as a predictor of intrinsic rewards and enjoyment*
- *The data files were moved to figshare and are openly available*
- *The manuscript was reformatted to LaTex*

The paper titled "Study of Enjoying Transnarrative Media." This paper investigates the concept of transnarrative media and how audiences engage with and enjoy these narratives. It aims to explore the characteristics of transnarrative media and the factors influencing audience enjoyment.

PONE-D-25-32038

Harvesting Easter Eggs: An Exploratory Study of Enjoying Transnarrative Media

PLOS ONE

Dear Dr. Watts,

Thank you for submitting your manuscript to PLOS ONE. After careful consideration, we feel that it has merit but does not fully meet PLOS ONE’s publication criteria as it currently stands. Therefore, we invite you to submit a revised version of the manuscript that addresses the points raised during the review process.

The topic is timely and creative, and both reviewers—along with myself—see clear potential in the contribution this study could make. At the same time, the manuscript will require substantial revision before it can progress further. As the reviewers and I noted, the literature review needs to be expanded to include more recent and foundational work on transnarrative media, narrative engagement, and audience motivations. Integrating research from areas such as TEBOTS, Retrospective Imaginative Involvement, and mental model scholarship (including the work of Busselle and Bilandzic) will provide your study a more solid theoretical grounding. I also agree with the reviewers that briefly acknowledging related theories—such as Social Comparison Theory, Goal Orientation Theory, Achievement Goal Theory, and Self-Efficacy Theory—would help place your chosen framework of Self-Determination Theory within a broader scholarly context.

*Response: Upon reviewing the feedback, we agree that situating our research more broadly across the various theoretical perspectives would help scholars who are unfamiliar with intertextuality better understand the significance and contributions of this study. In the manuscript, we make a point to tie SDT to RII and TEBOTS. We also provide more theoretical grounding in the media enjoyment section to discuss how media impacts the self/others more broadly by mentioning social comparison theory, goal orientation theory, and self-efficacy. We appreciate these thoughtful recommendations.*

The reviewers and I also felt that your discussion of the audience's motivations and behaviours needs further development. Clarifying what drives individuals to engage in transnarrative activities, and how these motivations relate to Easter egg hunting, will strengthen the conceptual clarity of the manuscript. The connection between these two concepts is currently implied but not fully articulated. In addition, the treatment of mental models would benefit from acknowledging their limitations, which will help present a more balanced argument.

*Response: We have significantly rewritten the audience motivations and behaviors section. Specifically, we connect parasocial relationships to both the tripartite model and SDT. We have reframed transnarrative behaviors per another reviewer comment as fan behaviors, as we thought this better described the type of behavior we were interested in investigating. We also connect fan behavior to the tripartite model and SDT.*

*The section about mental models was revised to discuss the limitations as well as the research conducted by Busselle and Bilandzic. We believe the revised paper is much more informative and clarifies mental model research for readers.*

Several conceptual distinctions also need clearer boundaries. In particular, differentiating transnarrative activity from general fan behaviour—and explaining how parasocial relationships may or may not function as a mechanism—will help avoid conceptual overlap. I encourage you to soften the language when making strong claims, particularly regarding necessary conditions, unless you present strong empirical support.

On the methodological side, I agree with the reviewers that additional transparency is needed. Please ensure that the OSF link is accessible, clarify whether participants were given a definition of “easter eggs", provide a clearer justification for your fan identification item, and explain whether your intrinsic reward scale fully captures the three core SDT needs. Reporting the full CFA model is also important given the number of new measures. More generally, please ensure that the interpretations in the manuscript align with the analyses. One reviewer noted—and I agree—that the relationship between easter egg hunting and enjoyment should be examined directly, since it is central to the aims of the study.

*Response: We agree that the analyses and results did not answer the research questions and hypotheses we set out to explore. In the revised paper, we have reanalyzed our hierarchical regressions by including easter egg recall into the analyses. Specifically, we conducted hierarchical regressions to examine predictors of intrinsic rewards and enjoyment. We used easter egg seeking behavior as the first step, and incorporated predictors (easter egg recall, PSR, fan behavior) to examine relationships to intrinsic rewards, and an additional step including intrinsic rewards to examine the predictors on enjoyment. Our updated analyses are included as Tables 3-4. We also reported the CFA results as Table 1.*

Finally, the reviewers and I encourage you to refine several portions of the writing for clarity and to provide more detailed participant characteristics, especially regarding age and media experience, as these factors may meaningfully shape transnarrative engagement.

*Response: During the R&R, we reviewed the paper to make changes throughout the document. Some of these changes involved cutting and synthesizing areas that were tangential to the study, while other areas of the paper involved incorporating a more substantial review of the existing and related research to easter eggs.*

Overall, despite the substantial revisions required, I believe—as do the reviewers—that this project has real promise. If you decide to resubmit, please include a detailed response letter outlining how each point has been addressed.

*Response: We are encouraged by these positive comments/reviews and hope the revised paper addresses any remaining concerns about the study.*

Please submit your revised manuscript by Dec 29 2025 11:59PM. If you will need more time than this to complete your revisions, please reply to this message or contact the journal office at plosone@plos.org. When you're ready to submit your revision, log on to https://www.editorialmanager.com/pone/ and select the 'Submissions Needing Revision' folder to locate your manuscript file.

Please include the following items when submitting your revised manuscript:

- A rebuttal letter that responds to each point raised by the academic editor and reviewer(s). You should upload this letter as a separate file labeled 'Response to Reviewers'.
- A marked-up copy of your manuscript that highlights changes made to the original version. You should upload this as a separate file labeled 'Revised Manuscript with Track Changes'.
- An unmarked version of your revised paper without tracked changes. You should upload this as a separate file labeled 'Manuscript'.

If you would like to make changes to your financial disclosure, please include your updated statement in your cover letter. Guidelines for resubmitting your figure files are available below the reviewer comments at the end of this letter.

If applicable, we recommend that you deposit your laboratory protocols in protocols.io to enhance the reproducibility of your results. Protocols.io assigns your protocol its own identifier (DOI) so that it can be cited independently in the future. For instructions see: <https://journals.plos.org/plosone/s/submission-guidelines#loc-laboratory-protocols>. Additionally, PLOS ONE offers an option for publishing peer-reviewed Lab Protocol articles, which describe protocols hosted on protocols.io. Read more information on sharing protocols at <https://plos.org/protocols?utm_medium=editorial-email&utm_source=authorletters&utm_campaign=protocols>.

Thank you again for submitting your work, and I look forward to seeing your revised manuscript.

With best regards,

Hamed Ahmadinia, Ph.D.
Erikoistutkija | Senior Researcher
Taloussosiologia | Economic Sociology
Sosiaalitieteiden laitos | Department of Social Research
Turun yliopisto | University of Turku
Suomi | Finland
hamed.ahmadinia@utu.fi
Academic Editor | PLOS ONE (Social & Behavioral Sciences)

Journal Requirements:

When submitting your revision, we need you to address these additional requirements.

1. Please ensure that your manuscript meets PLOS ONE's style requirements, including those for file naming. The PLOS ONE style templates can be found at

<https://journals.plos.org/plosone/s/file?id=wjVg/PLOSOne_formatting_sample_main_body.pdf> and

<https://journals.plos.org/plosone/s/file?id=ba62/PLOSOne_formatting_sample_title_authors_affiliations.pdf>

*Response: The revised paper, to our knowledge, follows the formatting requirements of PLOS one. We used the PLOS one template in Overleaf.*

2. In the ethics statement in the Methods, you have specified that verbal consent was obtained. Please provide additional details regarding how this consent was documented and witnessed, and state whether this was approved by the IRB

*Response: The consent was described erroneously. In the online study, participants were asked to click a button indicating their consent and agreement to participate or not and being removed from the study. Per our IRB rules, this type of consent process is described as “written” consent. The paper is updated accordingly.*

3. Please update your submission to use the PLOS LaTeX template. The template and more information on our requirements for LaTeX submissions can be found at <http://journals.plos.org/plosone/s/latex>.

*Response: As mentioned, in the revised submission, our papers uses the PLOS LaTex template provided in Overleaf.*

4. Thank you for uploading your study's underlying data set. Unfortunately, the repository you have noted in your Data Availability statement does not qualify as an acceptable data repository according to PLOS's standards.
At this time, please upload the minimal data set necessary to replicate your study's findings to a stable, public repository (such as figshare or Dryad) and provide us with the relevant URLs, DOIs, or accession numbers that may be used to access these data. For a list of recommended repositories and additional information on PLOS standards for data deposition, please see <https://journals.plos.org/plosone/s/recommended-repositories>.

*Response: We have uploaded our data to Figshare and provided a DOI link in the paper to access the dataset. https://doi.org/10.6084/m9.figshare.30933761*

5. Please include your full ethics statement in the ‘Methods’ section of your manuscript file. In your statement, please include the full name of the IRB or ethics committee who approved or waived your study, as well as whether or not you obtained informed written or verbal consent. If consent was waived for your study, please include this information in your statement as well.

*Response: The full name of the IRB committee (the Institutional Review Board under the Human Research Protection Program at the University of Kansas) is mentioned in the methods section.*

6. We note you have included a table to which you do not refer in the text of your manuscript. Please ensure that you refer to Table 1 in your text; if accepted, production will need this reference to link the reader to the Table.

*Response: All tables are referred to in the text of the revised manuscript.*

7. If the reviewer comments include a recommendation to cite specific previously published works, please review and evaluate these publications to determine whether they are relevant and should be cited. There is no requirement to cite these works unless the editor has indicated otherwise.

[Note: HTML markup is below. Please do not edit.]

Reviewers' comments:

Reviewer's Responses to Questions

**Comments to the Author**

1. Is the manuscript technically sound, and do the data support the conclusions?

The manuscript must describe a technically sound piece of scientific research with data that supports the conclusions. Experiments must have been conducted rigorously, with appropriate controls, replication, and sample sizes. The conclusions must be drawn appropriately based on the data presented.

Reviewer #1: Yes

Reviewer #2: Partly

2. Has the statistical analysis been performed appropriately and rigorously?

Reviewer #1: Yes

Reviewer #2: Yes

3. Have the authors made all data underlying the findings in their manuscript fully available?

The [PLOS Data policy](https://nam10.safelinks.protection.outlook.com/?url=http%3A%2F%2Ftrack.editorialmanager.com%2FCL0%2Fhttp%3A%252F%252Fwww.plosone.org%252Fstatic%252Fpolicies.action%2523sharing%2F1%2F010f019a850caf58-b7c47759-2c21-4c8d-b45c-de838dd862ea-000000%2FMMfFj2ObXzg8Ufh7zLAC_VRn4bVCSWJNdTfKtwlv_OI%3D237&data=05%7C02%7Cjudy.watts%40ku.edu%7C9195462892744ead86e508de25f76e2d%7C3c176536afe643f5b96636feabbe3c1a%7C0%7C0%7C638989942154086377%7CUnknown%7CTWFpbGZsb3d8eyJFbXB0eU1hcGkiOnRydWUsIlYiOiIwLjAuMDAwMCIsIlAiOiJXaW4zMiIsIkFOIjoiTWFpbCIsIldUIjoyfQ%3D%3D%7C0%7C%7C%7C&sdata=1yug1v5L5MmrwxfLQGdPQb2eQYEmsAdMANu6nbJQmZw%3D&reserved=0) requires authors to make all data underlying the findings described in their manuscript fully available without restriction, with rare exception (please refer to the Data Availability Statement in the manuscript PDF file). The data should be provided as part of the manuscript or its supporting information, or deposited to a public repository. For example, in addition to summary statistics, the data points behind means, medians and variance measures should be available. If there are restrictions on publicly sharing data—e.g. participant privacy or use of data from a third party—those must be specified.

Reviewer #1: Yes

Reviewer #2: No

4. Is the manuscript presented in an intelligible fashion and written in standard English?

PLOS ONE does not copyedit accepted manuscripts, so the language in submitted articles must be clear, correct, and unambiguous. Any typographical or grammatical errors should be corrected at revision, so please note any specific errors here.

Reviewer #1: Yes

Reviewer #2: Yes

5. Review Comments to the Author

Please use the space provided to explain your answers to the questions above. You may also include additional comments for the author, including concerns about dual publication, research ethics, or publication ethics. (Please upload your review as an attachment if it exceeds 20,000 characters)

Reviewer #1: The paper titled "Study of Enjoying Transnarrative Media." This paper investigates the concept of transnarrative media and how audiences engage with and enjoy these narratives. It aims to explore the characteristics of transnarrative media and the factors influencing audience enjoyment.
Summary of Key Points
The authors present a thoughtful examination of transnarrative media, emphasizing the role of audience engagement. However, several areas need improvement to enhance the clarity and depth of the research.

*Response: We thank Reviewer 1 for their time and attention attending to this manuscript. Please continue reading our responses below to their comments/recommendations.*

Strengths
1. Innovative Topic: The exploration of transnarrative media is both timely and relevant, particularly in the context of evolving media consumption patterns.

*Response: We agree and appreciate that others also find transnarrativemedia research increasingly relevant in today’s evolving media landscape.*

2. Theoretical Framework: The application of Self-Determination Theory (SDT) is a solid foundation for understanding audience motivation and enjoyment.
Areas for Improvement

1. Comprehensive Literature Review: The paper requires a more extensive literature review, particularly incorporating recent studies that address transnarrative media and audience engagement. This will provide a stronger theoretical context for the research.

*Response: Across Reviewer 1 and Reviewer 2’s comments, we realized that our literature review did not sufficiently incorporate more recent audience engagement concepts and research. We have addressed this throughout the paper, especially when discussing media based SDT theories/research as well as more recent research related to mental models and entertainment. We hope these changes provide the reader with a better understanding of the many varied ways audiences have the potential to engage with narratives.*

2. Integration of Additional Theories: While the authors utilize Self-Determination Theory, it would be beneficial to mention and discuss competitive theories such as Social Comparison Theory, Goal Orientation Theory, Achievement Goal Theory, and Self-Efficacy Theory. Integrating these theories can provide a more nuanced understanding of how audiences interpret their experiences with transnarrative media.

*Response: We appreciate this recommendation. We discuss these theories on p. 3 of the manuscript by connecting these theories to enjoyment and/or meeting one’s needs. Specifically, we say:*

*Scholars have identified various causes of enjoyment with many media theories focusing on enjoyment as the meeting of needs. For instance, media can meet needs by providing relevant others to compare oneself to (i.e. social comparison theory sit), or by helping viewers feel capable and in control (i.e. self-efficacy theory sit). Media enjoyment, particularly through interactive media, can also be a result of goal achievement, evoking feelings of competence (i.e. achievement goal theory sit, or allowing for the performance of ability (i.e. goal orientation theory sit).*

3. Audience Behaviors: The authors briefly touch on audience behaviors; however, there is a need for a more comprehensive explanation of the various interactions and drivers that impact audience behavior. A detailed exploration of these drivers will enhance the understanding of audience engagement and experience.

*Response: Our aim in the revised paper was to better introduce fan behaviors, rather than audience behaviors broadly as the former is related to the research we conducted. As such, in the revised paper, we introduce audience behaviors via the tripartite model (p. 5), and discuss the types of audience behaviors that were previously examined. We then pivot to discussing behaviors (i.e., fan behaviors) that are likely to result from exposure to transnarrative media. In sum, the paper explains audience behavior more in-depth, both how it has been operationalized in previous literature, as well as why it should be relevant to easter egg behavior specifically.*

4. Mental Models and Limitations: The authors state, “In the current study, we propose that the ability to construct mental models of transnarrative media results in enjoyment and an intrinsically rewarding experience.” It is essential to address the limitations associated with mental models, including:
• Simplification of Reality
• Bias and Subjectivity
• Rigidity (Resistance to Change)
• Cognitive Overload
Acknowledging these limitations will provide a more balanced perspective and enhance the credibility of the research.

*Response: The revised manuscript describes several criticisms from prior studies and academics about mental models theory, including ability to construct a mental model, simplification of reality, ability to recall mental models, and variation in individuals' mental model capabilities.*

5. Participant Characteristics: The paper should better address the characteristics of the participants, particularly their range of ages and experiences. This information is crucial for understanding how these factors may influence audience engagement and enjoyment.

*Response: The paper now includes all of the participant characteristics that were included in the study, included age range (18 – 78), the percentage and n of ethnic/race demographics, and education level.*

*Once again, we thank Reviewer 1 for their helpful and instructive feedback.*

Reviewer #2: I want to thank the editors of Plos One for the opportunity to review “Harvesting Easter Eggs: An Exploratory Study of Enjoying Transnarrative Media.” I also want to commend the authors for introducing and examining the concepts of transnarrative and easter egg hunting for entertainment scholars. While I don’t think the survey and findings are game changing (and that’s my personal opinion), I do think most of the flaws in this paper are addressable, and a version of this study should be published. I hope the feedback below is helpful for the authors in their future endeavors with this project.

*Response: We appreciate Reviewer 2’s insightful feedback and comments on improving the manuscript. While this study is more exploratory rather than groundbreaking, we hope that this study encourages other scholars to consider the connections between transnarrative media and enjoyment.*

1. On page 6, are the authors trying to distinguish between ADT of humor and ADT of drama (otherwise known as the moral sanction theory of delight and repugnance; Zillmann, 2000). This isn’t clear the way it is written currently.

*Response: We cut out unnecessary and extraneous content throughout, and other than acknowledging Zillmann’s contributions to entertainment research, ADT is not mentioned as a theoretical focus of the present research. Nabi. We see how bringing up ADT without exploring it further may be confusing or distracting, so we opted to remove the reference to ADT and transition directly into the media enjoyment perspectives we rely on throughout the paper. As such, we place a larger focus on Tamborini’s SDT approach to entertainment and Nabi and Krcmar’s tripartite model as the main theories guiding the research.*

2. On page 7, “However, it has been less frequently applied to traditional forms of entertainment, including movies and television shows.” There is work in The temporarily expanding boundaries of the self (TEBOTS) literature (e.g., Johnson et al., 2016) and retrospective imaginative involvement (RII) literature (e.g., Sherrick et al., 2022) that connects SDT intrinsic need fulfillment and narrative exposure and enjoyment. In fact, work in RII has a strikingly similar argument that ties narrative engagement and intrinsic satisfaction with the argument presented in this paper (i.e., the role of updating and expanding mental models). Incorporating the logic from that literature may be a useful consideration for this paper.

*Response: We thank the reviewer for noting that there are many connections between SDT and traditional forms of entertainment. Although these forms of entertainment differ from the one studied in our research, these are extremely relevant and worth noting. We mention RII, boundary expansion, and SDT on p. 3 of the revised manuscript.*

3. On pages 7 and 8, the introduction and discussion of Nabi and Krcmar’s enjoyment paper feel underdeveloped to me and a bit tacked on. I think the connecting logic that links the tripartite model to the other variables needs more work, because the explanation given feels vague. It is not enough to say that emotion, cognition, and behavior are correlated with enjoyment. What aspects of these broader categories are we pinpointing here, and how and why do they embody or cause enjoyment?

*Response: We developed the argument about Nabi And Krcmar's model (beginning on p. 4) and explained more clearly how it relates to easter eggs. We also strengthened the transition so it is more clear how the mechanisms we discuss later (PSR and fan behaviors) are extensions of this argument.*

*Specifically, we highlight this passage from our revised manuscript (p. 4)*

*The affective component includes feelings of empathy, as well as discrete emotions evoked by a narrative. Cognitive reactions include elaborating upon information within a narrative and making judgments. Behavioral elements include those that lead to viewing, occur during viewing, or take place after viewing sit [2]. These three responses are interrelated and together shape the multidimensional attitude that is media enjoyment. This perspective reflects what we anticipate to be true of easter eggs: the experience of discovering an easter egg should evoke an affective response, require cognitive engagement, and lead to behaviors such as information sharing and seeking further ways to engage in the media universe. More specifically, searching for and finding easter eggs should lead to feelings of excitement, anticipation, or frustration, and will require cognitive processing to search through potential symbols present in the narrative. Subsequently, the intertextual elements of transnarrative media could lead to in-viewing behaviors such as pausing, rewinding, or talking with others, as well as post-viewing behaviors such as talking to other fans in person or online, and engaging with fan forums.*

4. On page 8, I can see an underlying mechanism the drives transnarrative activities being PSR, i.e., the authors states, “This connection with characters is part of what drives viewers to participate in broader media universes.” However, as it is written now, I think this claim needs further explanation and evidence to support it.

*Response: We have considerably rewritten the subsection regarding PSR’s connection with transnarrative media and characters that span narratives. Additional explanation and support has been provided to strengthen this claim at the bottom of p. 4.*

5. On page 9, what makes transmedia activities different from fan behaviors? There seems to be a high level of conceptual overlap here with clear boundary conditions. One could say the motives are different, but at least from an operational standpoint, I’m not sure they are distinguishable. I bring this up in caution because we want to avoid an overproliferation of variables that could create confusion in the field.

*Response: This point is well taken, and we agree that while there are minor conceptual differences between the terms, using already established terms should be prioritized in order to maintain consistency in the field. Therefore, we renamed our "transnarrative behavior" variable "fan behavior," and specified how we would be using the term in a transnarrative context.*

6. On pages 9 and 10, it’s a bit odd to discuss the role of mental model in narrative engagement without citing and discussing Bussell and Bilandzic’s work. They set the theoretical groundwork to explain how audiences develop and expand story mental models and how the process of doing so impacts enjoyment. The authors need to incorporate this work in any future revision.

*Response: We agree with the reviewer that we left out some seminal research and theorizing about the connection between mental models and enjoyment. In the revised manuscript we summarize Busselle and Bilandzic's work on p. 6.*

7. On page 10, “When applying mental models to transnarrative media, it is probable that some audiences seek out easter eggs, while others are less motivated to find these hidden symbols.” This leads to the question of who and under what conditions. The authors have some idea of this, e.g., fans, which answers RQ1, but it isn’t set up here.

*Response: We made the connection clearer between the assertion that motivation and ability will vary across viewers, our literature-based assumptions about who will be high in motivation and ability, and our first research question. We, however, do not provide more specific predictions associations between which audiences or under what circumstances we expect audiences to seek easter eggs as this is beyond the scope of our exploratory study.*

8. After reading the front part of the paper, it isn’t fully clear in my mind how transnarrative behavior and easter egg hunting are connected theoretically. Intuitively, it makes sense, but it feels like the paper covers two parallel ideas that don’t cross. This is also reflected in the findings, where the authors don’t explore their link.

*Response: In the previous version of our paper, we only examined predictors of intrinsic rewards and enjoyment from those that recalled an easter egg. We understand that this was not ideal, while also not fully delving into the phenomena of easter egg exposure, discovery, and the psychological mechanisms audiences encounter. We re-analyzed our data using hierarchical regressions with the full dataset. In the revised analyses (pp. 11 – 12) we entered easter egg seeking behavior as the first step, and easter egg recall, PSR, and fan behavior (formerly called transnarrative behavior) as the second step to examine these as predictors to intrinsic rewards. We wanted to investigate in our updated analyses that beyond individuals who tend to look for easter eggs during their media exposure (easter egg seeking behavior), that actually finding an easter egg, the behaviors associated with them, and relationships to other characters predict the rewarding feeling we anticipated. Indeed, our results indicate that easter egg recall, PSR, and fan behavior predict intrinsic rewards. We carried out a similar process to examine these predictors, and intrinsic rewards, to entertainment.*

9. The OSF link doesn’t work (the repository is private).

*Response: Thank you for alerting us that the OSF link is not working. We have uploaded our data to Figshare per the editor’s comments.*

10. In the survey, did you provide a definition of easter eggs to your participants?

*Response: Yes, in the survey we provided a definition of easter eggs (see p. 7). We include the definition here as well.*

*We think of easter eggs as hidden or subtle messages in a movie/TV show that reference the story world, events, or characters from another movie/TV show. Sometimes these easter eggs reference elements from other stories told within the same universe, like a reference about events from Iron Man in a Thor movie. Other times easter eggs reference characters from the same production studio like Rapunzel appearing in the crowd at the end of Frozen. Easter eggs can occur in all sorts of movies/TV shows, and some viewers catch these subtle nods while others may not notice them at all.*

11. The authors should quote the fan item. It’s also a bit odd that a previous fandom scale wasn’t used.

*Response: In the revised manuscript we included a reference which was adapted from a validated scale. Vinney et al. 2019*

12. For the intrinsic reward scale, does it capture all three SDT motives? The example item is unclear whether it does. If not, that limits how the authors can interpret their findings in the discussion.

*Response: No, the scale used in our study more closely captures competence than the other SDT concepts. We note that our use of the intrinsic rewards measure was a limitation in the study. However, intrinsic rewards is an integral part of the SDT as it examines the motivating factors for individuals.*

13. The authors need to provide the full CFA model, especially given the number of new scales made. Also, while the first model isn’t great, its fit isn’t too bad either. So you may be a bit too harsh. I’m not a fan of correlating error terms within the same measure unless the items’ wording are nearly similar.

*Response: The factor loadings for the full CFA model are included in the manuscript. Only error terms that were correlated were the third and fourth items of parasocial relationships, the first and second items of intrinsic rewards, and the second and fourth items of enjoyment, all of which were similarly worded items.*

14. On page 15, the first sentence in the Validity Check section is grammatically incorrect. Also, the final sentence in the paragraph is a bit of an overclaim: The authors didn’t support mental model research (in my opinion) based on this data.

*Response: The first sentence in the validity check section was corrected and the last sentence was removed.*

15. I find it a bit odd that this paper wanted to examine if easter egg hunting contributes to audience enjoyment, but it never directly tests it. The authors have the data for this, so I don’t see why it isn’t reported.

*Response: As we mentioned in a prior response to the reviewer’s feedback, we have re-analyzed our results and included easter egg recall as a predictor. We thank the reviewer for raising this issue with us.*

16. On page 19, “Because parasocial relationships are associated with enduring imagined relationships with media personae and fictional characters, PSR may need to be established for the enjoyment and rewarding experience of transnarrative stories to occur.” This is a strong claim (i.e., setting up a necessary condition) that I don’t think is always the case. Other drivers besides PSR could drive transnarrative enjoyment, such as curiosity, a desire to world-build, or being in a fan community. I think this claim should be softened.

*Response: We agree with Reviewer 2 that there are likely many factors that contribute to an audience member’s transnarrative enjoyment. We have revised our discussion section to soften the language here.*

Again, I think this is an interesting study, but overall, some of the arguments need to be more concise, and the analyses should test the arguments presented in the paper.

*Response: We thank Reviewer 2 for their comments and feedback.*

6. PLOS authors have the option to publish the peer review history of their article ([what does this mean?](https://nam10.safelinks.protection.outlook.com/?url=https%3A%2F%2Ftrack.editorialmanager.com%2FCL0%2Fhttps%3A%252F%252Fjournals.plos.org%252Fplosone%252Fs%252Feditorial-and-peer-review-process%2523loc-peer-review-history%2F1%2F010f019a850caf58-b7c47759-2c21-4c8d-b45c-de838dd862ea-000000%2Fp2oBCRzUic6ml22h-neYz-f-iLhJZaSAM9zqFYA7Ukw%3D237&data=05%7C02%7Cjudy.watts%40ku.edu%7C9195462892744ead86e508de25f76e2d%7C3c176536afe643f5b96636feabbe3c1a%7C0%7C0%7C638989942154125114%7CUnknown%7CTWFpbGZsb3d8eyJFbXB0eU1hcGkiOnRydWUsIlYiOiIwLjAuMDAwMCIsIlAiOiJXaW4zMiIsIkFOIjoiTWFpbCIsIldUIjoyfQ%3D%3D%7C0%7C%7C%7C&sdata=%2FF1ZoBzJ2r5xPVYuX0HdPEf8YXW1IhQxZSYM1pxy3xA%3D&reserved=0)). If published, this will include your full peer review and any attached files.


If you choose “no”, your identity will remain anonymous but your review may still be made public.


**Do you want your identity to be public for this peer review?** For information about this choice, including consent withdrawal, please see our [Privacy Policy](https://nam10.safelinks.protection.outlook.com/?url=https%3A%2F%2Ftrack.editorialmanager.com%2FCL0%2Fhttps%3A%252F%252Fwww.plos.org%252Fprivacy-policy%2F1%2F010f019a850caf58-b7c47759-2c21-4c8d-b45c-de838dd862ea-000000%2FwWjJXgFOa8PuvTx9NliLf6AmTBJKfa82GrcdjyQLxx8%3D237&data=05%7C02%7Cjudy.watts%40ku.edu%7C9195462892744ead86e508de25f76e2d%7C3c176536afe643f5b96636feabbe3c1a%7C0%7C0%7C638989942154158287%7CUnknown%7CTWFpbGZsb3d8eyJFbXB0eU1hcGkiOnRydWUsIlYiOiIwLjAuMDAwMCIsIlAiOiJXaW4zMiIsIkFOIjoiTWFpbCIsIldUIjoyfQ%3D%3D%7C0%7C%7C%7C&sdata=fKRJKGRg3cODPkwtCvBkvJ9dHweutWGla62YrxKQI9U%3D&reserved=0).

Reviewer #1: **Yes**

Reviewer #2: No
